# Supplementary material for: NLRC3 Attenuates Antiviral Innate Immune Response by Targeting IRF7 in Grass Carp (Ctenopharyngodon idelus)
Source: Int J Mol Sci. 2025 Jan 20;26(2):840. doi: 10.3390/ijms26020840 (PMC11766192; doi:10.3390/ijms26020840)
Supplement: Supplementary file 1 [file ijms-26-00840-s001.zip › ijms-3440523-supplementary.pdf]

Supplemental Table S1. Primers used in this study

Primer name	Sequence (5′-3′)
NLRC3-siRNA-1	GAUACAUUGAGAUCCUGAA(dT)(dT)
NLRC3-siRNA-1	UUCAGGAUCUCAAUGUAUC(dT)(dT)
NLRC3-siRNA-2	CAAGUAAGCUAGCACAGAA(dT)(dT)
NLRC3-siRNA-2	UUCUGUGCUAGCUUACUUG(dT)(dT)
β-actin-qR	GGGCACCTGAACCTCTCATT
β-actin-qF	GCTATGTGGCTCTTGACTTCG
GCRV-P4-F	GCGACAAAGACTGGCTGCGG
GCRV-P4-R	GAGGCACAGGTTGTCGAGGG
GCRV-P5-F	CTTCTGCCTAAGCCCAAGCG
GCRV-P5-R	GGGAGGGCAGGATAGTGAGAC
GCRV-P6-F	CACTTGGCGAGACCCAGTCC
GCRV-P6-R	CGGTACGCCAGTGTATGCCA
CI-IFN-QF	GGCCGATACAGGATGATAAGC
CI-IFN-QR	GCATCCATGAGGCGGATGATA
CI-MX-QF	AGAACTGAAAGAGCGACTGGAG
CI-MX-QR	TACAGAATGCACAAACTTGGAC
ISG15-qF	GGTGGTGAAAGTTGATGCCA
ISG15-qR	CGTTGACCGTTGTCGCTAGA
ISG20-qF	TACACTGCCTGGTGAAGCTG
ISG20-qR	ATTCCCACACATGGCGAACT
CiNLRC3-QF	ATTCTCTGGGGATGGACGGA
CiNLRC3-QR	TGTACCAGCTTTGATGGCGT
NLRC3-R-GFP	TGGTGGCGGCGGATCCGATATCCACCACACAATCT GGTGTACC
cherry-IRF3-F	TCTTTTTGCAGGATCCATGACCCATCCAAAACCGCT
cherry-IRF3-R	CGAATCGATGGGATCCCACTTGGTGTCACACAACTC CA
cherry-IRF7-F	TCTTTTTGCAGGATCCATGGCAGCGATGCAGAGC
cherry-IRF7-R	CGAATCGATGGGATCCGAGTCCATTGAAGGCAGAC CC
CiNLRC3-HindIII	GTTTAAACTTAAGCTTATGGCTAGACATGACCATAT CCCATG
CiNLRC3-BamHI	CTGGACTAGTGGATCCGATATCCACCACACAATCTG GTGTACC
